# Removal of AU Bias from Microarray mRNA Expression Data Enhances Computational Identification of Active MicroRNAs

**DOI:** 10.1371/journal.pcbi.1000189

**Published:** 2008-10-03

**Authors:** Ran Elkon, Reuven Agami

**Affiliations:** Division of Gene Regulation, The Netherlands Cancer Institute, Amsterdam, The Netherlands; Weizmann Institute of Science, Israel

## Abstract

Elucidation of regulatory roles played by microRNAs (miRs) in various biological networks is one of the greatest challenges of present molecular and computational biology. The integrated analysis of gene expression data and 3′-UTR sequences holds great promise for being an effective means to systematically delineate active miRs in different biological processes. Applying such an integrated analysis, we uncovered a striking relationship between 3′-UTR AU content and gene response in numerous microarray datasets. We show that this relationship is secondary to a general bias that links gene response and probe AU content and reflects the fact that in the majority of current arrays probes are selected from target transcript 3′-UTRs. Therefore, removal of this bias, which is in order in any analysis of microarray datasets, is of crucial importance when integrating expression data and 3′-UTR sequences to identify regulatory elements embedded in this region. We developed visualization and normalization schemes for the detection and removal of such AU biases and demonstrate that their application to microarray data significantly enhances the computational identification of active miRs. Our results substantiate that, after removal of AU biases, mRNA expression profiles contain ample information which allows in silico detection of miRs that are active in physiological conditions.

## Author Summary

MicroRNAs are a novel class of genes that encodes for short RNA molecules recognized to play key roles in the regulation of many biological networks. MicroRNAs, predicted to collectively target more than 30% of all human protein-coding genes, suppress gene expression by binding to regulatory elements usually embedded in the 3′-UTRs of their target mRNAs. Despite intensive efforts in recent years, biological functions carried out by microRNAs have been characterized for only a small number of these genes, making elucidation of their roles one of the greatest challenges of biology today. Bioinformatics analyses can significantly help meet this challenge. In particular, the integrated analysis of microarray mRNA expression data and 3′-UTR sequences holds great promise for systematic dissection of regulatory networks controlled by microRNAs. Applying such integrated analysis to numerous microarray datasets, we disclosed a major technical bias that hampers the identification of active microRNAs from mRNA expression profiles. We developed visualization and normalization schemes for detection and removal of the bias and demonstrate that their application to microarray data significantly enhances the identification of active microRNAs. Given the broad use of microarrays and the ever-growing interest in microRNAs, we anticipate that the methods we introduced will be widely adopted.

## Introduction

MicroRNAs (miRs) are a growing class of non-coding RNAs that is now recognized as a major tier of gene control, predicted to target more than 30% of all human protein-coding genes [1],[2]. miRs suppress gene expression via binding to regulatory sites usually embedded in the 3′-UTRs of their target mRNAs, leading to the repression of translation occasionally associated with mRNA degradation. Target recognition involves complementary base pairing of the target site with the miR's seed region (positions 2–8 at the miR's 5′ end), although the exact extent of seed complementarity is not precisely determined, and can be modified by 3′ pairing [2]–[4]. Despite intensive efforts in recent years, biological functions carried out by miRs have been characterized for only a minority of these genes, and therefore, elucidating regulatory roles played by miRs in various biological networks constitutes one of the major challenges facing biology today. Bioinformatics analyses can significantly contribute to elucidation of miR functions; in particular, the integrated analysis of gene expression data and 3′-UTR sequences that holds promise for systematic dissection of regulatory networks controlled by miRs and of cis-regulatory elements embedded in 3′-UTRs.

Similar bioinformatics approaches that integrates gene expression data and promoter sequences proved highly effective in delineating transcriptional regulatory networks in a multitude of organisms ranging from yeast to human [5]–[7]. Microarray measurements reflect the total effect of all regulatory mechanisms that control gene expression, including both transcriptional and post-transcriptional mechanisms; thus, genome-wide expression profiles should yield ample information not only on transcriptional networks, but also on regulatory networks regulated by miRs and RNA binding proteins (RBPs) that modulate mRNA stability, and that usually act via regulatory elements in 3′-UTR of their target genes [8]. Although mRNA degradation seems to be a secondary mode of miRs' action (with inhibition of translation being the primary one), since each miR is predicted to directly affect the expression level of dozens of target genes, such an orchestrated effect should be discernable by statistical analysis of wide-scale mRNA expression data, even if the effect on each target is only a subtle one. This orchestrated effect could serve as a molecular fingerprint for miRs activity under given biological conditions. Indeed, several pioneering studies provided strong evidence of the ability to computationally decipher miR-mediated regulatory networks from mRNA expression data alone or in correlation with miR expression profiles [9]–[14].

In this study, we applied an integrated analysis of gene expression data and 3′-UTR sequences aimed at identifying miRs that are active in a given biological process. Applying such analysis we discovered in numerous microarray datasets a major bias that resulted in a striking relationship between 3′-UTR AU content and gene response. We show that this surprising link between gene's response and 3′-UTR base composition is secondary to a more basic relationship between gene's response and base composition of its probes on the chip. We demonstrate that this bias causes many false positive calls in computational searches for active miRs from mRNA expression data. Therefore, removal of this bias, which is in order in any analysis of microarray datasets, is of crucial importance when integrating expression data and 3′-UTR sequences to identify regulatory elements embedded in this region. Our results substantiate that computational analysis of mRNA expression data, after appropriate removal of AU biases, can accurately detect active miRs that control various biological processes under physiological conditions.

## Results

We set out to demonstrate that integrated computational analysis of mRNA expression data and 3′-UTR sequences can accurately uncover miRs that participate in the regulation of a given biological process. As the role of miRs in different branches of hematopoiesis is well characterized [15]–[18], we first analyzed a dataset that recorded global gene expression profiles for multi-potent hematopoietic progenitor cells (HPCs) undergoing multi-lineage differentiation [19]. Since miRs often induce degradation of their target mRNAs, we expected the 3′-UTR of genes whose expression is induced during differentiation to be enriched for seed signatures of miRs that become inactive in this process, and vice versa—that the 3′-UTR of genes whose expression is repressed would be enriched for seed signatures of miRs that become active during the process.

Before employing statistical tests to identify over-represented seed sequences among up- or down-regulated genes, we examined whether a more global trend in base composition could be detected in the 3′-UTR sequences of the responding genes. For example, if the 3′-UTRs of the up-regulated genes are generally more AU-rich compared to the 3′-UTRs of the non-responding genes, then any statistical search for over-represented seed signatures among the up-regulated genes is expected to yield false positive calls for miRs whose seed signature is AU-rich. One effective means for detecting such false positive calls is to repeat the over-representation tests with randomly permuted miR seeds (which preserve the seed's base composition). If an enrichment of a certain miR seed is accounted for merely by base composition, then it is expected to be non-specific and detected also for randomly permuted seeds derived from the original one.

**Figure 1:**
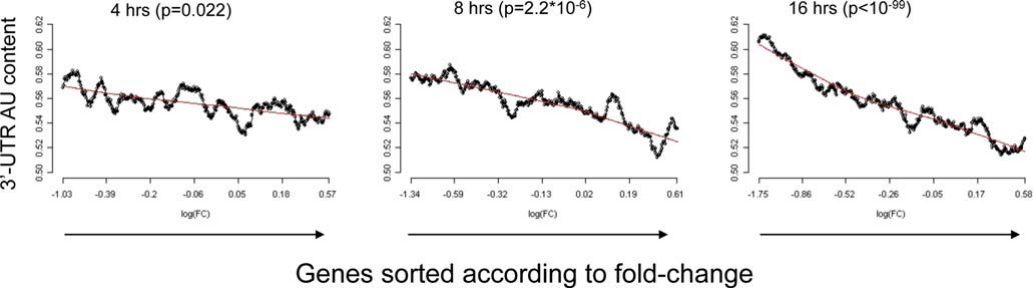
Relationship between 3′-UTR AU content and gene response during HPC differentiation. Expression profiles were measured at several time points after stimulation of HPC differentiation into megakaryocytes. To visualize the relationships between 3′-UTR AU content and gene response, the genes were sorted for each time point according to their fold of repression/induction relative to the expression level at t0, and the mean 3′-UTR AU content was calculated in a sliding window that encompassed in each step 5% of the genes included in the analysis. (At each step the sliding window was moved to the right by 5% of its size.) Each plot corresponds to the time point indicated above it. Genes are sorted on the X-axis according to their response, from the most repressed genes at the left to the most induced genes at the right. The Y-axis represents the mean 3′-UTR AU content calculated on each sliding window. The *p* value above each plot is for the comparison (Wilcoxon test) between the 3′-UTR AU content of the top 5% (most strongly up-regulated) and bottom 5% (most strongly down-regulated) genes at the corresponding time point. Note the striking relationship between 3′-UTR AU content and gene response at the 16 hr time point.

Therefore, as a first step in the analysis of the HPC dataset, we checked whether a global 3′-UTR base composition trend is associated with the multi-lineage differentiation. We detected a very strong correlation between 3′-UTR base composition and gene response at several time points in this dataset. For example, there was an exceptionally strong relationship between AU content and gene response at the 16 hr time point after induction of HPC differentiation into megakaryocytes: 3′-UTRs of down-regulated genes were significantly more AU-rich than those of up-regulated ones (Figure 1). (The mean 3′-UTR AU content of the 5% most down-regulated and most up-regulated genes were 60.6% and 52.7%, respectively, *p*<10^−99^, Wilcoxon test.) The other three lineages in this dataset displayed similarly strong trends (Figure S1).

**Figure 2:**
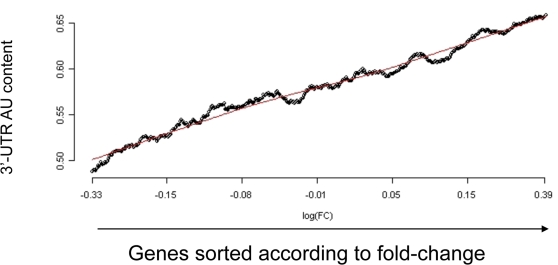
Strong relationship between 3′-UTR AU content and gene response detected in a comparison between technical replicates. The figure shows the relationship between 3′-UTR AU content and gene fold-change in a comparison between two chips hybridized with identical universal reference RNA pools. The plot was generated as described in the legend to Figure 1. A highly significant relationship between 3′-UTR AU content and gene response was detected in this technical comparison (*p* value = 8.1*10^−84^ for the comparison between the bottom and top 5% ‘responding’ genes), pointing to a major AU bias in microarray measurements.

The strength of the relationship between 3′-UTR AU content and gene response in the HPC dataset prompted us to search for such trends in other datasets. Surprisingly, we found such relationships, with similarly high statistical significance, in numerous microarray datasets (data not shown). Still more suspicious, we observed the relationship even when we compared different control samples within a dataset. This led us to question whether the relationship observed between 3′-UTR AU content and gene response reflects any true biological regulatory mechanism, or is rather a result of some technical artifact in microarray measurements. We found a definitive answer to this question by analyzing a technical dataset published by van Ruissen et al. [20]. This dataset profiled a universal reference RNA pool in two independent oligonucleotide chips (Affymetrix HGU133A). Comparing the data from these two arrays, which measure identical and artificial RNA pools, we again found a striking relationship between 3′-UTR AU content and difference in gene expression level (Figure 2), pointing to a major AU bias in microarray measurements. This AU response bias is not specific to a particular data preprocessing method, as it existed in data under different preprocessing and normalization schemes; namely, rma, gcrma, and mas5 (Figure S2). In this technical dataset, we detected no preference for A or U in the bias, and no major 3′-UTR length bias (Figure S3).

**Figure 3:**
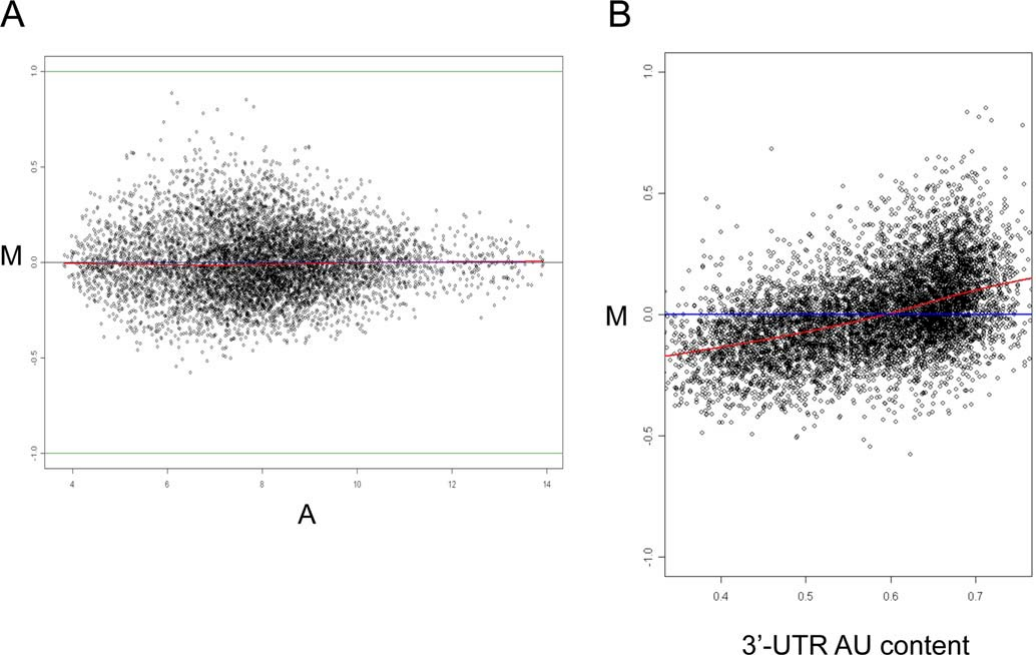
M-A and M-AU plots. (A). M-A plot shows that there is no intensity-response bias in the comparison between the two chips hybridized with identical universal reference RNA pools. The Y axis (denoted as M) represents the log2 fold-change and the X-axis (denoted as A) represents the average log2 intensity. Each dot in the plot corresponds to a gene in the dataset. (B). Adopting the M-A plot concept, we introduced the M-AU plot, in which the Y axis represents the log2 fold-change (as in the M-A plots), and the X axis represents the 3′-UTR AU content of a gene. The M-AU plot shows a major AU bias in this technical dataset. The red line is the lowess smoothing line calculated for the scatter plot.

Next, we sought to elucidate the sources of the AU response bias. A well-documented bias in microarray measurements is the one between probe intensity and response [21], which is routinely visualized using M-A plots. We first suspected that the observed association between 3′-UTR AU content and gene response is a mere reflection of the intensity-response bias. However, there was no intensity-bias in the above technical dataset, which points that the 3′-UTR AU response bias is distinct from the intensity-response bias (see Figure 3A and 3B; in the latter, adopting the concept of M-A plots, we introduced the M-AU plot to visualize the AU response bias). The AU response bias exists over a large range of intensities (Figure S4), and, furthermore, the gcrma method which takes into account the correlation between probe's AU content and intensity did not cancel it.

**Figure 4:**
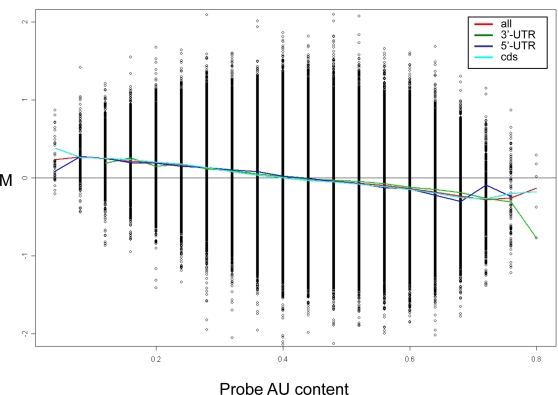
The AU response bias is related to probe base composition regardless probe location along the target transcript. Probe-level M-AU plot for the comparison between two chips hybridized with a common human brain reference sample. This dataset used the new generation Affymetrix Human Gene 1.0 ST Array, in which probes are located throughout the target transcripts. We generated plots which either included all probes, or included separately only those mapped to the 5′-UTR, CDS, or 3′-UTR of the targets. (As the length of each probe is 25 bases, probe's AU content (X axis) gets only discrete values in the 0–100% range with jumps of 4%). Probes mapped to the different transcript regions exhibited similar level of AU response bias.

In the vast majority of present chips, probes are selected from the 3′-end of target transcripts. This is also the case for the technical dataset that we have analyzed, which used the Affymetrix HGU133A chip. Therefore, as expected, we observed in this dataset also a strong relationship between probeset AU content and response (similar to the one observed between gene's 3′-UTR AU content and response) (Figure S5). To test whether the AU artifact origins either from base-composition properties of 3′-UTR of target transcripts or of that of the chip probes, the sequence of probes and target 3′-UTRs need to be uncoupled. The new generation Affymetrix chips break this coupling as their probes are selected from all regions of target transcripts. We therefore analyzed a second technical dataset, recently published by Pradervand et al. [22] which used the new Affymetrix Human Gene 1.0 ST Array. In this dataset too, we detected a strong AU response bias. That is, we observed a significant relationship between probeset AU content and response in a comparison between duplicate control chips. Importantly, carrying out a probe-level analysis, we found that probes located at 5′-UTR and CDS regions show a similar AU bias as probes located at 3′-UTRs (Figure 4). This finding indicates that the link between gene's response and 3′-UTR base composition is secondary to a more basic bias in microarray measurements which links gene response with base composition of its probes.

We next evaluated the effect of the AU bias on computational identification of active miRs from microarray data. Searching for miRs that are active in biological conditions examined in a dataset, we utilized miR target prediction generated by TargetScanS [2], and applied the following statistical test: for each miR family and for each condition in a dataset, we tested whether the set of predicted miR target genes is significantly induced or repressed compared to a background set consisting of all the non-target genes (see Methods). The technical dataset which profiled the universal reference RNA pool served us as a negative test case in which no real biological signal exists. Applying the statistical tests to this dataset, we identified nine miR families whose target sets showed statistically significant response (Table 1). Of course, in this negative test case, all calls are false positive ones; and, as expected, all the falsely identified miR families had an AU-rich seed (the seed of eight out of the nine calls contained at least 5 A or U bases, while the prevalence of miRs with such seed among all the miRs tested was less than 25%; Table 1). Next, for each miR family identified as significant, we repeated the statistical tests, but this time with randomly permuted miR seeds. In all cases, permuted seeds showed similar statistical significance to the original seeds (Table 1), demonstrating the utility of such permutation tests in detecting non-specific results caused by correlation between base composition of miR-seeds and 3′-UTRs of the responding genes.

**Table 1:** Active miRs falsely identified in the negative test case.

Without AU Normalization			
miR ID	*p* Value	miR Seed	Best Permuted *p* Value^a^
miR.186	2.41*10^−9^	AAAGAAU	1.60*10^−11^
miR.543	1.72*10^−7^	AACAUUC	2.01*10^−7^
miR.496	2.24*10^−7^	UUACAUG	3.14*10^−7^
miR.200b.429	3.07*10^−7^	AAUACUG	9.84*10^−10^
miR.381	1.41*10^−5^	AUACAAG	2.48*10^−10^
miR.26	1.62*10^−5^	UCAAGUA	4.07*10^−5^
miR.203.1	1.79*10^−5^	GAAAUGU	6.34*10^−6^
miR.132.212	0.00017	AACAGUC	0.0019
miR.181	0.00029	ACAUUCA	3.45*10^−10^

After AU Normalization^b^			
miR ID	*p* Value	Best Permuted *p* Value^a^	
miR.186	0.0061	0.0060	
miR.500	0.0073	0.0025	

a Best *p*-value obtained for 20 randomly permuted seeds derived from the original miR seed.

b After applying AU normalization to the dataset none of the miRs passed the statistical significance threshold (0.0003, which corresponds to 0.05 after Bonferroni correction for multiple testing). In order to compare the results with the original data (without AU normalization), we listed the top two miRs even though they did not pass the threshold.

**Figure 5:**
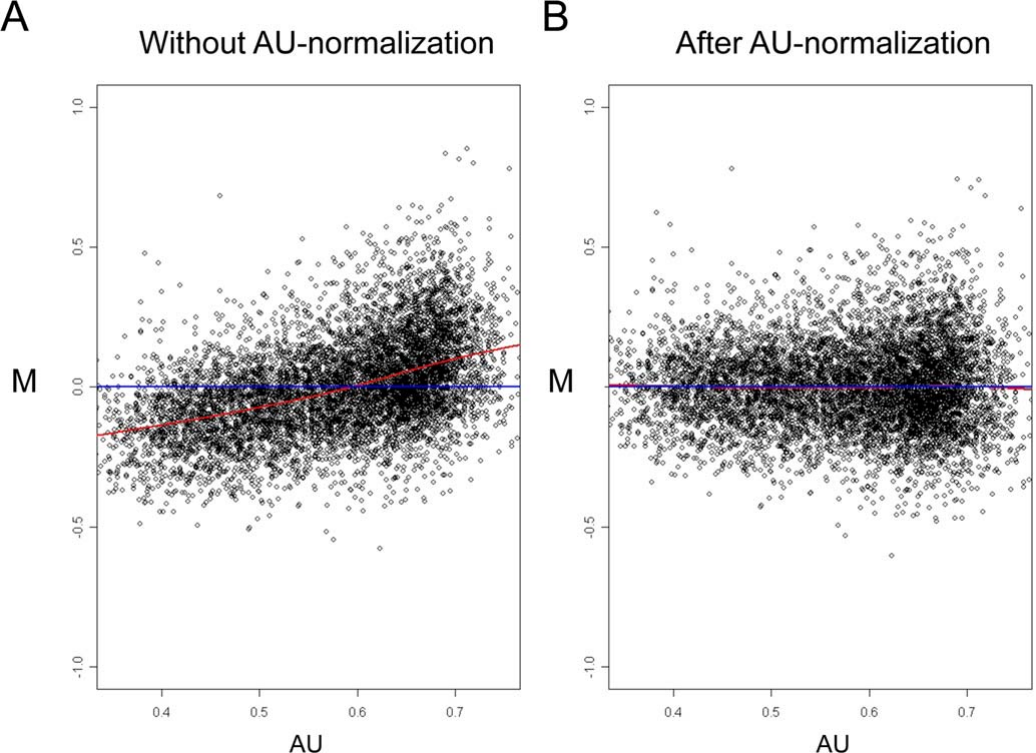
AU normalization. M-AU plots without (A) and after (B) applying an AU normalization scheme to the technical dataset which profiled the universal reference RNA pool.

As shown, the AU response bias causes many false positive calls in computational search for active miRs from expression data, and therefore its removal is crucial when carrying out integrated bioinformatics analysis of mRNA expression data and 3′-UTR sequences. To remove this bias, we adopted the *lowess* normalization method which is routinely used to remove intensity biases from microarray data [21], and adjusted it to cancel AU biases (Figure 5) (see Methods). Applying AU normalization did not distort the normalization at the M-A plane (Figure S6). Importantly, after applying AU normalization to the negative control dataset, no miR family passed the statistical significance threshold (0.0003, which corresponds to 0.05 after Bonferroni correction for multiple testing) (Table 1).

**Figure 6:**
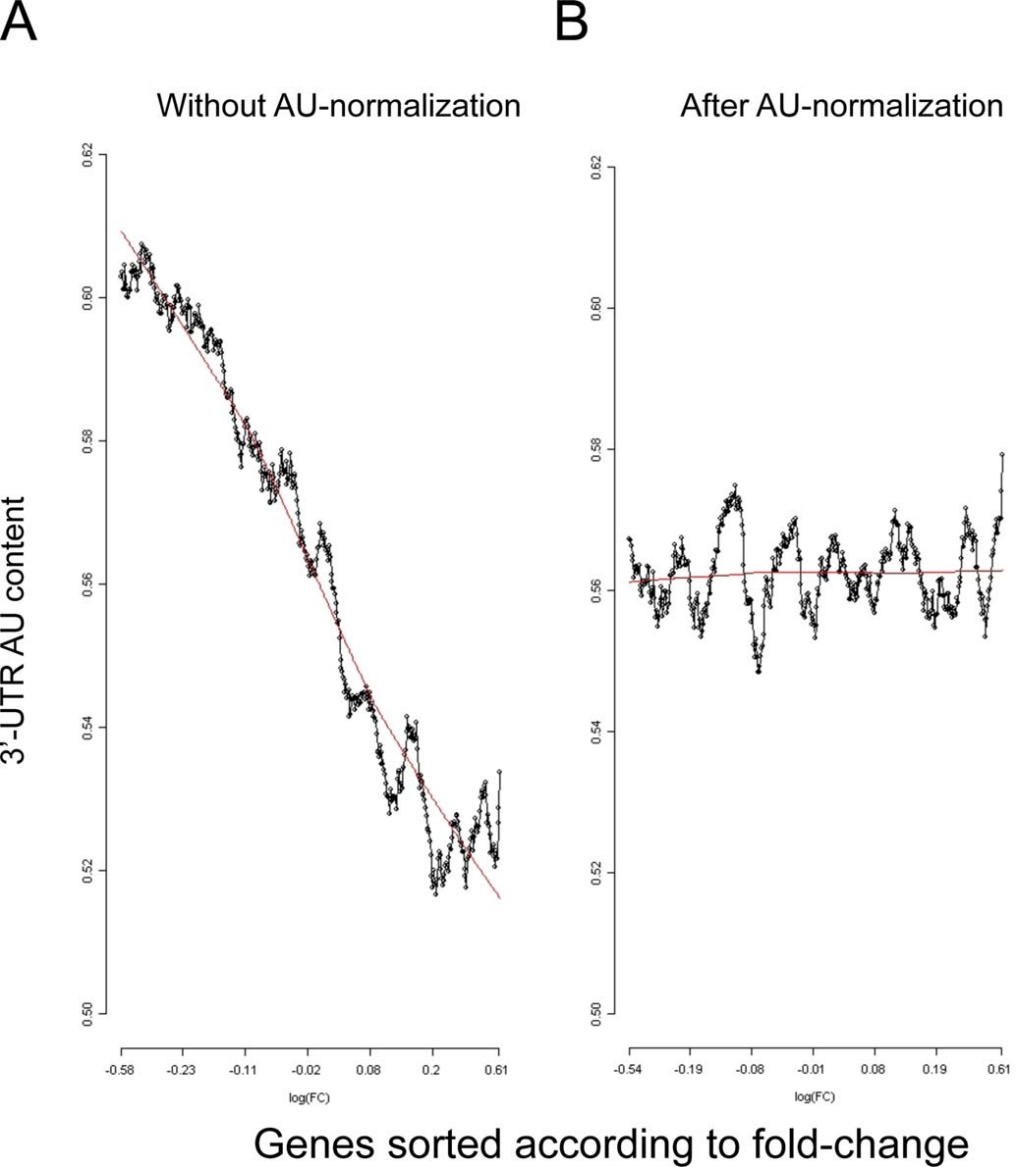
AU bias in the miR-155 dataset. Relationship between 3′-UTR AU content and gene response in the dataset that compared gene expression profiles between miR-155-deficient and control Th2 cells. (A) Without AU normalization. (B) After applying AU normalization to the dataset. Plots were generated as described in the legend to Figure 1.

We next searched for an expression dataset that would serve as a positive test case; that is, a dataset that contains known miR signals. We preferred physiologically relevant datasets over ones that over-expressed miRs, which often give expression levels that are far above physiological ones. (Statistical searches for active miRs applied to several datasets that profiled cells over-expressing specific miRs readily detected the correct signals both without and after AU normalization (data not shown).) A recent study that compared expression profiles between stimulated T-cells derived from miR-155 deficient and control mice met this requirement [23]. As in many other datasets, we observed a strong AU bias in this dataset too, and removed it using the AU normalization (Figure 6). Without AU normalization, the statistical tests identified eleven significant miR families; the true hit (miR-155) was the third most significant one (Table 2). (Note that five out of the six most significant miRs falsely identified on the negative dataset were detected also in this positive dataset (compare Tables 1 and 2)). Here too, permutation tests found, in most cases, random seeds whose significance scores were similar to the ones obtained by the original seeds (Table 2). In sharp contrast, after AU normalization, only the true miR (miR-155) was detected and its statistical significance was substantially improved (Table 2). Importantly, none of the permuted seeds derived from the seed of miR-155 obtained a statistically significant score.

**Table 2:** Active miRs identified in the miR-155 dataset.

Without AU Normalization		
miR ID	*p* Value^a^	Best Permuted *p* Value^b^
miR.496	−1.56*10^−10^	−1.13*10^−7^
miR.186	−1.43*10^−08^	−2.29*10^−9^
***miR.155***	7.07*10^−08^	−3.98*10^−6^
miR.26	−2.15*10^−06^	−1.41*10^−7^
miR.543	−2.33*10^−06^	−6.04*10^−6^
miR.25.32.92.363.367	−6.54*10^−06^	−3.71*10^−7^
miR.381	−1.09*10^−05^	−9.99*10^−8^
miR.329	−1.98*10^−05^	−1.31*10^−3^
miR.331	2.48*10^−05^	2.19*10^−1^
miR.493.5p	−3.98*10^−05^	−1.46*10^−10^
miR.495	−7.41*10^−05^	−9.60*10^−8^

After AU Normalization^a^		
miR ID	*p* Value	Best Permuted *p* Value^b^
***miR.155***	1.20*10^−12^	−0.023
miR.142.5p	0.00083	−0.024

a The sign of the *p*-value marks the direction of the response of the miR target set: positive and negative *p*-values correspond to miRs whose target sets are significantly up- and down-regulated in miR-deficient Th2 cells, respectively, compared to wild type Th2 cells. The results obtained for the true signal in this dataset—miR-155—are emphasized in bold-italic font, and are in the expected direction: that is, the set of miR-155 predicted target genes is up-regulated in miR-155 deficient Th2 cells compared to control Th2 cells.

b Best *p*-value obtained for 20 randomly permuted seeds derived from the original one.

For a more challenging test case we used a dataset that monitored gene expression profiles in five distinct human T cells sub-populations representing five phases of T cell differentiation [24]: intrathymic T progenitor (ITTP) cells, double positive (DP) thymocytes, CD4 single positive (SP4), naïve CD4 T cells from cord blood (CB4), and naïve CD4 T cells from adult blood (AB4). To obtain fold-change measures, we divided the expression level at each development phase by the one measured in the mature AB4 T cells. Without AU normalization, the statistical tests identified six significant miR families: the target sets of three were down-regulated in ITTP cells, and the target sets of the other three were up-regulated in the SP4 cells (Table 3). After applying the AU normalization to the data, only the three miR-families whose target sets were repressed in ITTP (miR-17.5p, miR-19 and miR-181 families) remained significant (Table 3), suggesting that members of these three miR families are active in early phases of T cell development and become inactive as T cells mature. There is evidence that all three miR families detected by the statistical analysis play a role in thymocyte maturation and therefore are true hits. Li et al. recently [25] showed that miR-181a is highly expressed in immature T cells and that its expression level goes down as T cells proceed through differentiation. That study further showed that miR-181a plays a critical role in augmenting T cell sensitivity, a propensity that is vital to the elimination of self-reacting T cells early during maturation. Regarding miR-17.5p and miR-19 families, Landais et al. recently reported that the miR-106-363 cluster is over-expressed in 46% of human T-cell leukemias tested [26]. The miR-106-363 cluster is homolog to the miR-17-92 cluster, and miR-19 is contained in both clusters but carries a seed which is different from the one of the other miRs in these two clusters. It is possible that up-regulation of members of the miR-106-363 and miR-17-92 clusters in T-cell leukemia endows these cells with propensities normal to immature T-cells, most probably enhanced proliferation capacity. The identification of true hits on this dataset further demonstrates that computational analysis can accurately dissect active miRs from gene expression data probing cells under physiological conditions. Our statistical analysis utilizes target prediction based on miR seed signatures and therefore cannot distinguish between miRs sharing seed sequences. Empirical biological testing is required to pinpoint which members of the miR-17-92 and miR-106-363 clusters that carry a common seed sequence are actually active during T cell maturation.

**Table 3:** Active miRs identified in the thymocyte maturation dataset.

Original Dataset				
miR ID	ITTP	DP4	SP4	CB4
miR.17.5p.20.93.mr.106.519.d	−1.45*10^−9^	−0.0055	−0.12	−0.75
miR.19	−7.04*10^−8^	−0.0085	−0.52	−0.040
miR.101	0.76	0.0059	1.27*10^−6^	−0.97
miR.144	0.84	0.0048	1.48*10^−6^	0.48
miR.381	0.40	0.0064	3.81*10^−5^	0.77
miR.181	−5.30*10^−5^	−0.649	0.12	−0.35

After Applying AU Normalization				
miR ID	ITTP^a^^#^	DP4	SP4	CB4
miR.17.5p.20.93.mr.106.519.d	−1.04*10^−9^ (−4.27*10^−3^)	−0.0016	−0.053	0.99
miR.19	−2.72*10^−8^ (−1.24*10^−2^)	−0.0010	−0.20	−0.08
miR.181	−3.19*10^−5^ (−7.07*10^−4^)	−0.20	0.43	−0.67

a In parentheses, the best *p*-value in 20 random seed permutations.

## Discussion

In the course of this study we observed in many gene expression datasets a striking association between gene response and 3′-UTR base composition. The high prevalence of such a relationship in microarray datasets, its exceptional statistical strength, and its detection in technical comparisons between replicate arrays, point unequivocally to a major bias in microarray measurements that was heretofore missed. Such a major AU bias in microarray measurements might have gone undetected because gene expression data are commonly analyzed in association with promoter, rather than 3′-UTR sequences, in attempts to unravel cis-regulatory promoter elements that control gene transcription. Only recently, with the emergence of miRs and RNA-binding proteins as key post-transcriptional regulators of gene expression, has gene expression analysis been coupled with analysis of 3′-UTR sequences. Indeed, it was the search for active miRs that motivated us to integrate gene expression and 3′-UTR sequence data, and led us to the detection of the AU response bias in microarray data.

We demonstrated that this bias is distinct from the well-documented intensity-response and AU intensity biases, and that it originates from a systematic association between probe base composition and response. Using the new generation Affymetrix chips that contain probes selected throughout the transcripts, we uncoupled the sequences of probes and target 3-UTRs. We show that probes exhibit similar AU response bias irrespective of their location in the target transcripts. Therefore, the major link between gene response and 3′-UTR base composition that we observed in vast microarray datasets, is secondary to the general probe AU response bias, and simply reflects the fact that chip probes were selected from 3′-UTRs. A reasonable explanation to the AU response bias is that there are subtle differences in hybridization conditions for different arrays in a dataset, and that the effect of such differences is dependent on probe base composition. Further technical examinations are required to test this point.

Bioinformatics analysis that integrates gene expression data and 3′-UTR sequences holds promise for systematic dissection of regulatory networks controlled by miRs. However, we demonstrated that the AU response bias causes many false positive calls in such analysis. Permutation tests were highly effective in revealing such false positive hits. Removal of this bias is of crucial importance when aiming to uncover miR-signatures as well as other cis-regulatory elements embedded in 3′-UTRs from mRNA expression profiles. We therefore developed visualization and normalization schemes for the detection and removal of AU biases, and demonstrated that their application to microarray data significantly enhances the computational identification of active miRs. In the case of Affymetrix chips, the normalization scheme that we implemented works at the probe-set or transcript level, and corrects the AU bias in a post-processing step (i.e., ran after probe intensity levels were calculated). A normalization scheme that takes into account the AU response bias at the phase of probe intensity calculation (similar to gcrma, which cancels AU intensity biases) is still required.

Our results further substantiate that mRNA expression data contain ample information that allows, after proper removal of AU biases, in silico detection of active miRs. Importantly, this is also true when mRNA profiles were measured under physiological conditions. In view of the importance of elucidating regulatory roles played by miRs in various biological networks, we anticipate that the methods introduced in this study for detection, visualization and removal of the AU response bias from microarray data will be in wide use by the research community.

## Methods

All statistical analyses were performed and plots were generated using the R package (http://www.r-project.org/).

### Data Analysis of Gene Expression Datasets

In this study, we analyzed four microarray datasets which used 3′-UTR Affymetrix oligonucleotide chips (that is, chips in which probes are selected from targets' 3-UTRs), and one dataset that used the new generation Affymetrix Human Gene 1.0 ST Array, in which probes are located throughout the target transcripts. Raw data files (CEL files) were downloaded from GEO (http://www.ncbi.nlm.nih.gov/geo/) or ArrayExpress (http://www.ebi.ac.uk/microarray-as/aer/#ae-main0) DBs, or obtained directly from the authors of the data.

#### Analysis of datasets that used 3′-UTR Affymetrix chips

The dataset that profiled HPC multi-lineage differentiation [19] used Affymetrix MGU74Av2 mouse chips. Expression levels were recorded in triplicates at 0, 4, 8, 16, 24, 48, 72, and 168 hrs of differentiation into four lineages: megakaryocytes, neutrophils, erythrocytes and macrophages. The dataset that profiled Stratagene's universal human reference RNA pool in two independent chips ([20], GSE1158) used Affymetrix HGU133A human chips. The dataset that profiled expression levels in miR155-deficient and control T cells ([23], E-TABM-232), used Affymetrix MG-430.2 mouse chips. Expression levels were measured in 5 replicates in miR155-deficient and wild-type Th1 and Th2 cells stimulated for 24 hrs with LPS and IL4. The results reported in our study were derived from the Th2 dataset. The dataset that profiled expression level during T cell maturation ([24], GSE1460), used Affymetrix HGU133A-B human chips. Expression levels were recorded in triplicates in 5 phases during differentiation (intrathymic T progenitor (ITTP) cells, double positive (DP) thymocytes, CD4 single positive (SP4), naïve CD4 T cells from cord blood (CB4), and naïve CD4 T cells from adult blood (AB4).

All these four datasets were processed by a similar scheme: First, probeset expression levels were calculated using the rma, gcrma, and mas5 methods implemented in the affy [27] and gcrma packages of the BioConductor project [28]. Unless otherwise stated, results reported in this paper are the ones obtained using the rma method. Similar results were obtained for data processed by the mas5 and gcrma methods. Second, probeset presence flags were calculated using the mas5calls function implemented in the affy package, and probesets that got more ‘Absent’ calls than a certain threshold were removed from subsequent analysis. (Thresholds for the number of ‘Absent’ calls were: 18 (out of 30 chips) in the HPC differentiation into megakaryocytes dataset; one (out of 2 chips) in the universal RNA pool dataset; 3 (out of 10 chips) in the miR-155 dataset; and 10 (out of 18 chips) in the T cell maturation dataset.) Next, probesets were mapped to their corresponding genes using annotation files provided by Affymetrix, and in cases where a gene was represented by several probesets, we used the measurements of the probeset with the highest median intensity level. Intensity levels over replicate chips were averaged.

#### Analysis of the dataset that used the Affymetrix Human Gene 1.0 ST Array

CEL files of this dataset ([22], GSE9819) were downloaded from GEO, and probe-set expression values were calculated using rma. In this dataset, we detected significant 3′-UTR bias in a comparison between two chips hybridized with a common Ambion Human Brain Reference RNA pool (sample ids GSM247680 and GSM247680). Probe-level intensities were extracted using the pm function implemented by the affy package. Probes' sequences and genome coordinates were obtained from chip annotation files provided by Affymetrix. Genome coordination of 5′-UTR, CDS and 3′-UTR regions of all annotated human transcripts were extracted from Ensembl using BioMart utilities [29]. Mapping of probes to 5′-UTR, CDS, and 3′-UTR regions was done by a Perl script written for this purpose. Before generating the probe-level M-AU plot, we performed the following preprocessing steps: a floor cut-off signal, which was set to the first quartile signal, was applied to each chip; probe expression levels were quantile- normalized; and probes whose signal was above median level were flagged as ‘Present’. Log2 of fold-change and AU content were calculated for each probe. To reduce noise, M-AU plot included only probes that were ‘Present’ in at least one of the chips hybridized with the brain reference sample.

### 3′-UTR Sequences and miR Target Prediction

3′-UTR sequences and miR target prediction for human and mouse were downloaded from TargetScanS (http://www.targetscan.org/; version 4.0; July 2007). TargetScanS predicts gene targets of miRNAs by searching 3′-UTRs for the presence of conserved 8-mer and 7-mer sites that match the seed region of each miRNA family [2]. In case a gene has several annotated 3′-UTRs, the longest one is considered.

#### Target prediction for randomly permuted miR seeds

For each conserved miR family, as defined by TargetscanS, we generated 20 randomly permuted seeds derived from the original seed. Targets of these random seeds were predicted by the same program used by TargetScanS for prediction of targets of the original miRs (the program is available at TargetScanS website).

#### AU normalization

Adopting the concepts of MA-plots and intensity-dependent normalization that were introduced by Yang et al. [21] in order to remove intensity biases from microarray data, we used the robust scatter plot smoother ‘lowess’, implemented in R (with default parameters), to remove the AU bias:
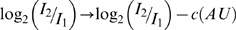
where *I_1_* and *I_2_* are the intensity signals measured for a gene in chip1 and chip2, and *c*(*AU*) is the lowess fit to the M-AU plot (in which the X-axis represents either transcript 3′-UTR, probe-set, or probe's AU content). Applying 3′-UTR-based or probe-set-based AU normalization to the 3′-UTR Affymetrix datasets yielded similar results, as expected, because of the coupling between transcript 3′-UTR and probe-set sequences in these chips.

#### Statistical search for candidate active miRs in mRNA expression dataset

Searching for miRs that are active in a microarray dataset, we utilized miR target prediction produced by TargetScanS, and applied the following statistical test: for each miR family and for each condition in a dataset, we tested whether the set of predicted miR target genes is significantly more induced/repressed than the background set consisting of all the non-target genes (for which 3′-UTR sequence and expression data are available). Target and background sets were compared using the non-parametric Wilcoxon test, and a miR family was putatively considered ‘active’ in a certain condition if the *p*-value obtained for its target set was below 0.05 after applying Bonferroni correction for multiple testing (∼150 miR families were tested).

## Supporting Information

Figure S1 Relationship between 3′-UTR AU content and gene response during HPC differentiation. The plot was generated as described in the legend to Figure 1 and shows the relationship between 3′-UTR AU content and gene response at three time points (4, 8, and 16 h) during HPC differentiation into three lineages (erythrocytes (E), monocytes (M), and neutrophils (N)).
(0.13 MB TIF)

Figure S2 AU bias in microarray data is not specific to a particular preprocessing method. The major AU bias in the dataset that profiled the universal reference RNA pool is not specific to a particular preprocessing method as it existed in data derived using different preprocessing and normalization schemes: rma, gcrma, and mas5.
(0.12 MB TIF)

Figure S3 No preference for A or U in the AU bias. The figure shows the relationship between gene fold-change in the technical dataset and: 3′-UTR AU content, 3′-UTR length, and 3′-UTR single base contents. The figure was generated as described in the legend to Figure 1 (*p* values indicated above each plot are for the comparison between the top 5% and bottom 5% genes). In this dataset, there is no preference for A or U in the relationship between 3′-UTR AU content and gene response. No major relationship between 3′-UTR length and gene response was observed here.
(0.13 MB TIF)

Figure S4 The AU response bias exists over large range of intensities. To test whether the AU-response bias is confined to probes with low intensities (which are inherently noisier), we redrew the M-A plot in Figure 3A, and colored each point according to the AU content of the corresponding probe (probes were divided into three groups: High, Medium and Low AU content probes; each group contained one third of the probes included in the analysis). The AU response bias is not associated with low intensity but exists over a large range of intensities.
(0.33 MB TIF)

Figure S5 AU bias using probe-set AU content. M-AU plot in which the X-axis represents probe-set AU content (in contrast to transcript 3′-UTR AU content shown in Figure 3B).
(0.13 MB TIF)

Figure S6 AU normalization does not distort the normalization at the M-A plane. This figure presents the M-A plot after applying AU normalization. While this normalization cancels the major bias detected at the M-AU plane, it has only subtle effect on the M-A plane.
(0.18 MB TIF)
