# Supplementary figures and images for: Removal of AU Bias from Microarray mRNA Expression Data Enhances Computational Identification of Active MicroRNAs
Source: PLoS Comput Biol. 2008 Oct 3;4(10):e1000189. doi: 10.1371/journal.pcbi.1000189 (PMC2533120; doi:10.1371/journal.pcbi.1000189)

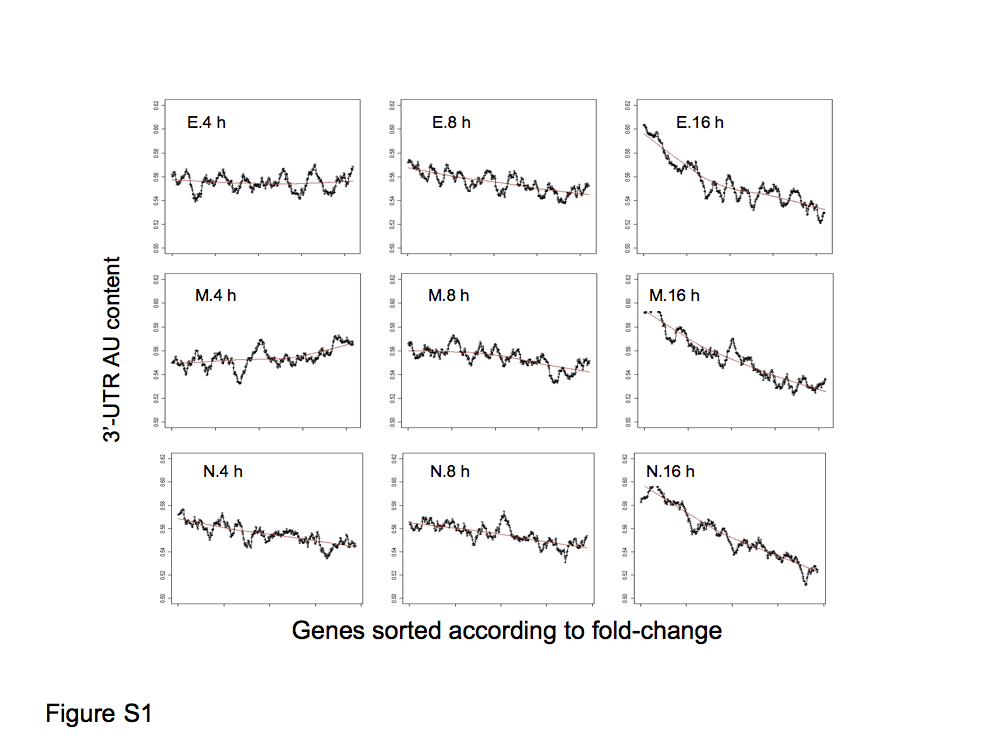

Supplement: Figure S1 — Relationship between 3′-UTR AU content and gene response during HPC differentiation. The plot was generated as described in the legend to Figure 1 and shows the relationship between 3′-UTR AU content and gene response at three time points (4, 8, and 16 h) during HPC differentiation into three lineages (erythrocytes (E), monocytes (M), and neutrophils (N)). (0.13 MB TIF) [file pcbi.1000189.s001.tif]

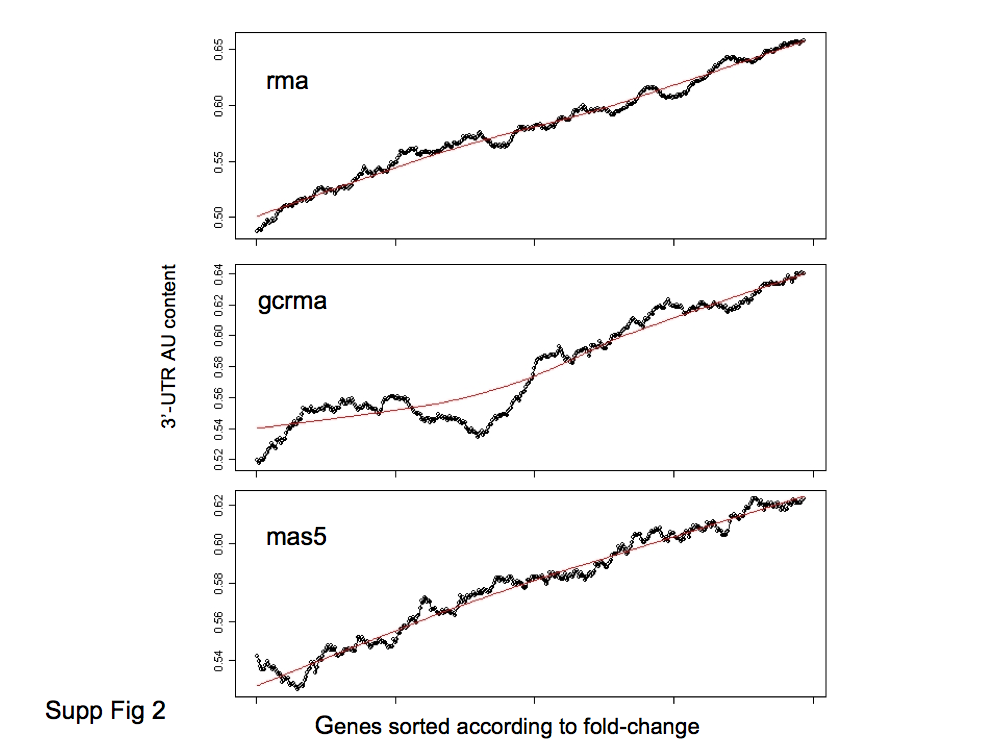

Supplement: Figure S2 — AU bias in microarray data is not specific to a particular preprocessing method. The major AU bias in the dataset that profiled the universal reference RNA pool is not specific to a particular preprocessing method as it existed in data derived using different preprocessing and normalization schemes: rma, gcrma, and mas5. (0.12 MB TIF) [file pcbi.1000189.s002.tif]

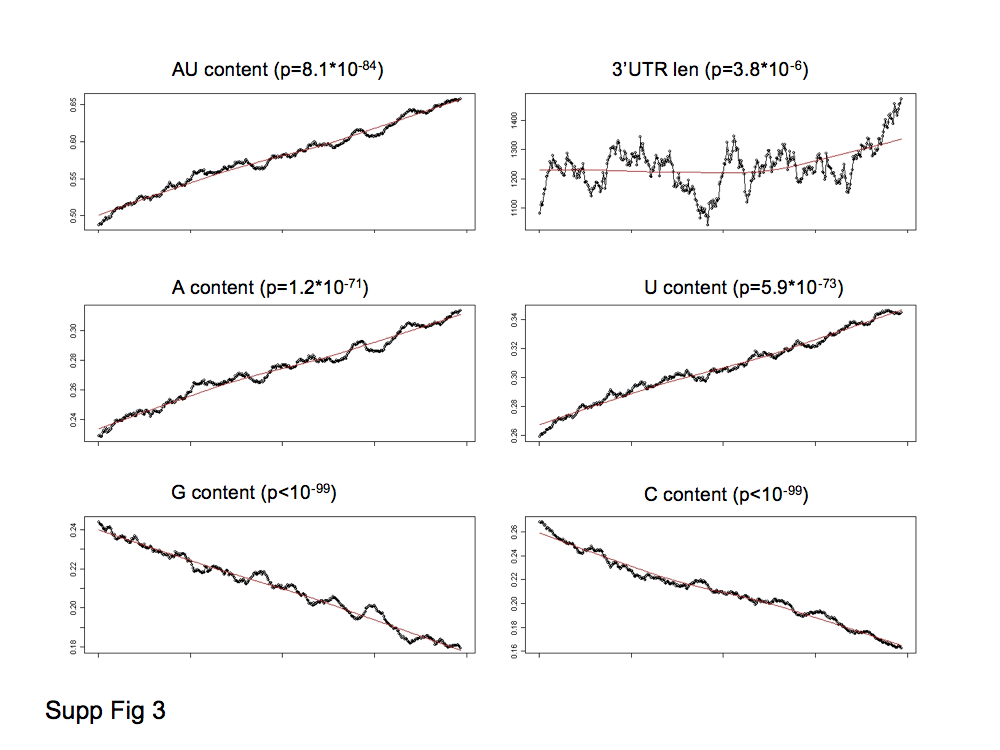

Supplement: Figure S3 — No preference for A or U in the AU bias. The figure shows the relationship between gene fold-change in the technical dataset and: 3′-UTR AU content, 3′-UTR length, and 3′-UTR single base contents. The figure was generated as described in the legend to Figure 1 (p values indicated above each plot are for the comparison between the top 5% and bottom 5% genes). In this dataset, there is no preference for A or U in the relationship between 3′-UTR AU content and gene response. No major relationship between 3′-UTR length and gene response was observed here. (0.13 MB TIF) [file pcbi.1000189.s003.tif]

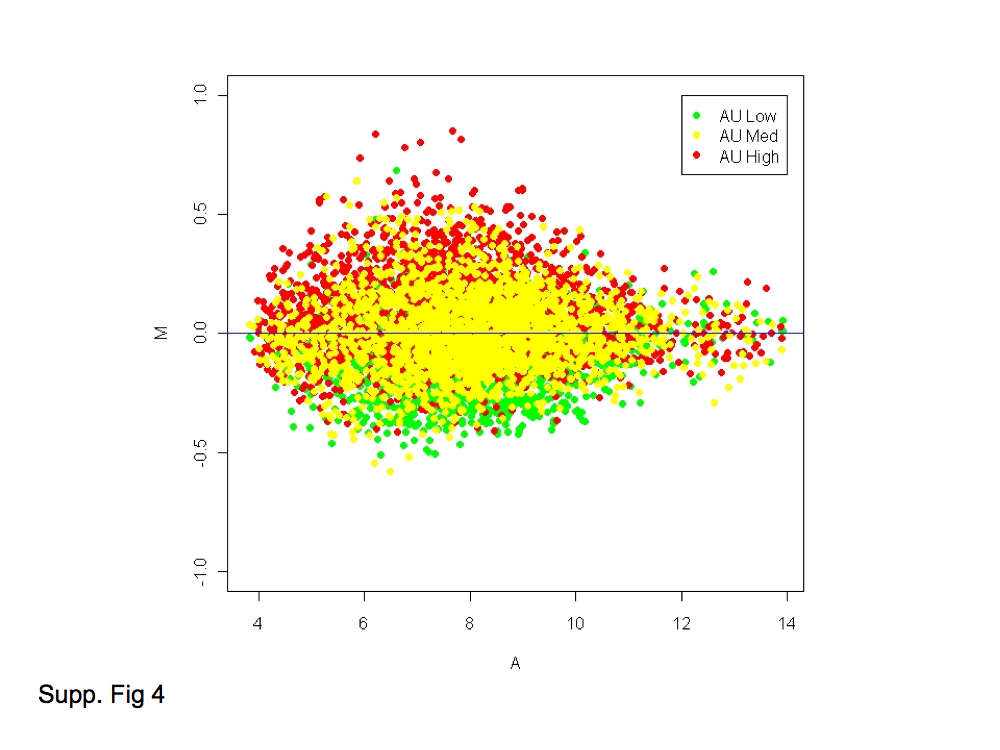

Supplement: Figure S4 — The AU response bias exists over large range of intensities. To test whether the AU-response bias is confined to probes with low intensities (which are inherently noisier), we redrew the M-A plot in Figure 3A, and colored each point according to the AU content of the corresponding probe (probes were divided into three groups: High, Medium and Low AU content probes; each group contained one third of the probes included in the analysis). The AU response bias is not associated with low intensity but exists over a large range of intensities. (0.33 MB TIF) [file pcbi.1000189.s004.tif]

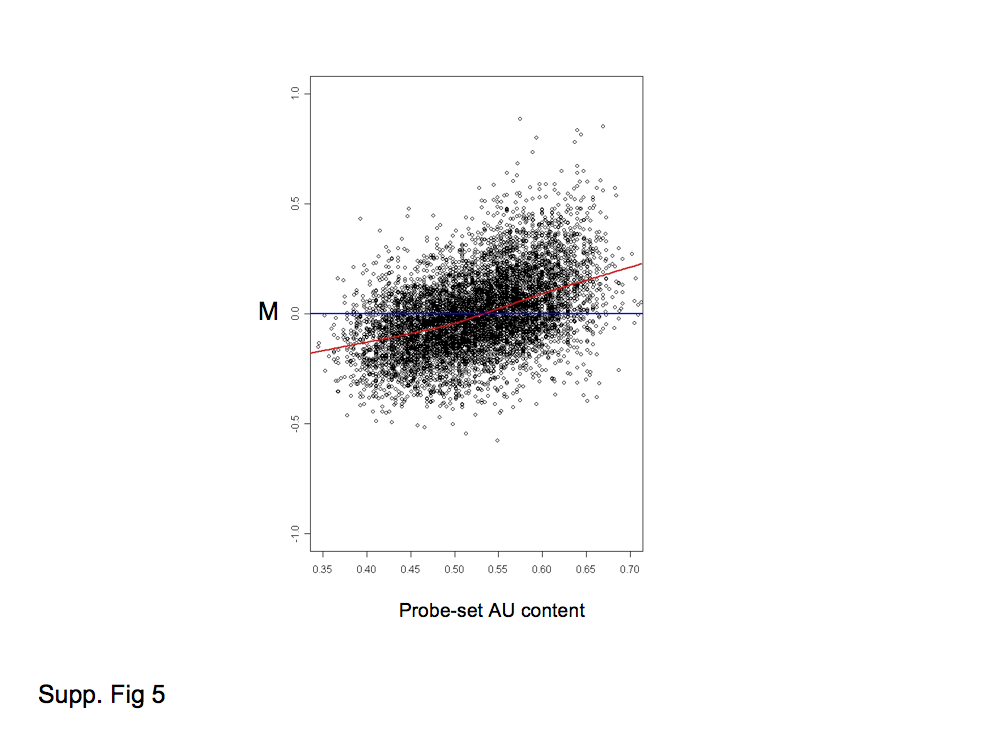

Supplement: Figure S5 — AU bias using probe-set AU content. M-AU plot in which the X-axis represents probe-set AU content (in contrast to transcript 3′-UTR AU content shown in Figure 3B). (0.13 MB TIF) [file pcbi.1000189.s005.tif]

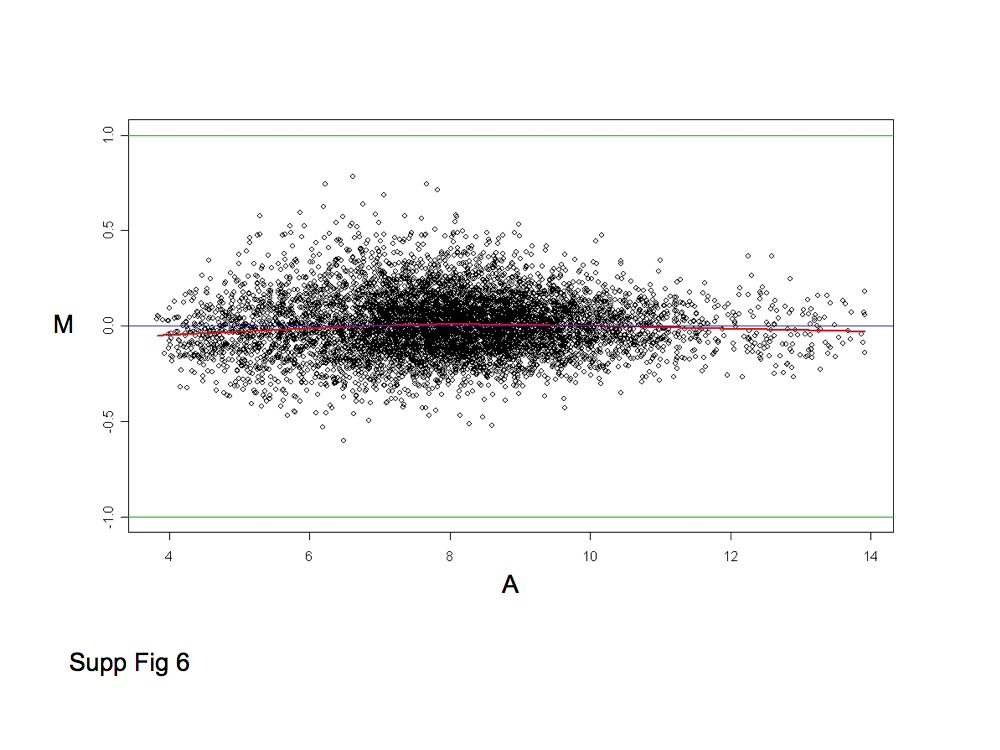

Supplement: Figure S6 — AU normalization does not distort the normalization at the M-A plane. This figure presents the M-A plot after applying AU normalization. While this normalization cancels the major bias detected at the M-AU plane, it has only subtle effect on the M-A plane. (0.18 MB TIF) [file pcbi.1000189.s006.tif]
